# Juvenile dermatomyositis in Latvia: clinical, radiologic, laboratory, and therapeutic findings from 2010 to 2025

**DOI:** 10.3389/fped.2026.1880638

**Published:** 2026-08-21

**Authors:** Lilija Banceviča, Zane Dāvidsone, Kristīna Budarina, Kristīne Rasa

**Affiliations:** 1 Faculty of Residency, Riga Stradins University, Riga, Latvia; 2 Department of Pediatrics, Riga Stradins University, Riga, Latvia; 3 Department of Pediatric Rheumatology, Children’s Clinical University Hospital, Riga, Latvia

**Keywords:** autoantibodies, autoimmune inflammatory myopathy, juvenile dermatomyositis, Latvia, pediatric rheumatology, treatment outcomes

## Abstract

**Background:**

Juvenile dermatomyositis (JDM) is a rare autoimmune inflammatory myopathy characterized by inflammation of skeletal muscle and skin, with potential multisystem involvement. Long-term population-based data remain limited, particularly in small countries such as Latvia. This retrospective analysis (2010–2025) describes epidemiological, clinical, laboratory, and imaging findings among children with JDM in Latvia.

**Materials and methods:**

We conducted a single-center retrospective case series of all children diagnosed with JDM at the Children’s Clinical University Hospital, Riga, between 2010 and 2025. Diagnosis was established according to the 2017 EULAR/ACR classification criteria. Clinical features, laboratory parameters, imaging findings, treatment strategies, and outcomes were analyzed.

**Results:**

Seventeen children were identified (58.8% female), with a median age at diagnosis of 6 years (range, 2–16 years). The mean annual incidence was 0.32 per 100,000 children. Disease was most frequently diagnosed in winter (52.9%). The median time from symptom onset to diagnosis was 90 days. The most common clinical features were symmetric proximal muscle weakness (70.6%), generalized weakness (70.6%), and characteristic skin manifestations (58.8%), including heliotrope rash and Gottron’s papules. Elevated lactate dehydrogenase (LDH) and erythrocyte sedimentation rate (ESR) were observed in 94% of children, whereas creatine phosphokinase (CPK) elevation occurred in 35%. Muscle magnetic resonance imaging demonstrated inflammatory changes consistent with myositis when clinically indicated. One child with JDM developed interstitial lung disease, one had gastrointestinal vasculitis, and one had clinically amyopathic JDM. All children received pharmacological treatment, most commonly glucocorticosteroids and non-steroidal anti-inflammatory drugs, with methotrexate used as the principal steroid-sparing agent. Remission within the first year was achieved in 70.6% of children using conventional therapy alone. Two children received biologic therapy. No mortality or macrophage activation syndrome was observed.

**Conclusions:**

This retrospective study covering 2010–2025 shows that JDM in Latvia generally presents similarly to that described in other cohorts of children. Proximal muscle weakness and characteristic skin findings were common, while CPK elevation was observed in only a subset of children. Most children responded well to treatment with glucocorticosteroids and methotrexate, with remission achieved in the majority within the first year and only rare need for biologic therapy. Muscle MRI was particularly helpful in selected cases when laboratory findings were inconclusive. Overall outcomes were favorable, with few severe complications and no mortality.

## 1 Introduction

Juvenile dermatomyositis (JDM) is an inflammatory muscle disorder characterized by mononuclear cell infiltration of striated muscle. Genetic predisposition is well recognized, particularly in association with certain human leukocyte antigen (HLA) types. Activation of the complement system contributes to microangiopathy through deposition of the membrane attack complex within vessel walls. The seasonal pattern observed in some cohorts has led to the hypothesis that infections may influence immune tolerance and trigger autoimmune responses. Proposed infectious triggers include Parvovirus B19, Coxsackievirus, Streptococcus, and various enteroviruses. Several non-infectious factors, including silica exposure, silicone implants, and lipid-lowering medications, have also been implicated. A strong association has been reported between JDM and the HLA-DR3 allele (1, 2).

To support a standardized diagnostic approach, the European League Against Rheumatism and the American College of Rheumatology (EULAR/ACR) developed classification criteria for idiopathic inflammatory myopathies, including JDM. The criteria use a weighted scoring system incorporating clinical findings, laboratory abnormalities, and muscle biopsy features when available (1, 3). The resulting score estimates the probability of inflammatory myopathy and allows classification as possible, probable, or definite disease. Importantly, the criteria can be applied without muscle biopsy, which is particularly relevant in children in whom characteristic skin manifestations may guide diagnosis. The classification algorithm can then identify the specific subtype, including JDM, in children with compatible clinical and cutaneous features.

Type I interferon (IFN) signaling is recognized as an important component of JDM pathogenesis. Increased expression of interferon-stimulated genes has been demonstrated in blood and muscle, reflecting activation of the IFN pathway (2, 4). This signaling contributes to muscle inflammation and vasculopathy and may amplify immune responses through effects on T cells, B cells, and dendritic cells.

The incidence and clinical expression of JDM vary across regions, yet data from Latvia remain limited. To date, no comprehensive overview of JDM presentation, disease course, management, or outcomes has been published from Latvia. This gap prompted us to review JDM cases diagnosed at the national tertiary pediatric referral center and characterize the local clinical experience with this rare autoimmune condition.

## 2 Materials and methods

We retrospectively reviewed all children diagnosed with JDM at the Children’s Clinical University Hospital, Latvia’s sole tertiary pediatric referral center, between January 2010 and December 2025. Diagnosis was established according to the 2017 EULAR/ACR classification criteria for adult and juvenile idiopathic inflammatory myopathies (1, 3). Clinical, laboratory, imaging, treatment, and outcome data were extracted from electronic medical records, including hospitalization and follow-up reports. None of the children had received immunosuppressive therapy before diagnosis. Annual incidence was calculated using data from the national health statistics database. Ethical approval was obtained from the Research Ethics Committee of Riga Stradins University (approval number 2-PĒK-4/125/2026; 18 December 2025).

## 3 Results

### 3.1 Demographic and epidemiological characteristics

Between January 2010 and December 2025, a total of 17 children were diagnosed with JDM at the Children’s Clinical University Hospital in Riga, Latvia. Of these, 10 (58.8%) were female and 7 (41.2%) were male, resulting in a female-to-male ratio of 1.4:1. The median age at diagnosis was 6 years (range, 2–16 years). The mean annual incidence of JDM during the study period was 0.32 per 100,000 children (Table 1). Disease onset occurred most frequently during winter months (52.9%), followed by spring (23.5%) and summer (23.5%); no new cases were recorded in autumn. The median duration of symptoms before diagnosis was 90 days, indicating a moderate diagnostic delay consistent with the rarity and heterogeneous presentation of the disease.

**Table 1:** Annual number of children diagnosed with juvenile dermatomyositis (JDM), child population in Latvia, and estimated annual JDM incidence per 100,000 children, 2010–2025.

Year	Number of children diagnosed with JDM	Child population in Latvia	Annual incidence per 100,000 children
2010	1	353,357	0.3
2011	1	360,216	0.3
2012	0	332,979	0.0
2013	1	347,018	0.3
2014	0	325,425	0.0
2015	0	348,660	0.0
2016	0	330,414	0.0
2017	1	356,527	0.3
2018	0	338,102	0.0
2019	2	358,813	0.6
2020	3	340,806	0.9
2021	2	352,800	0.7
2022	2	358,534	0.6
2023	1	356,864	0.3
2024	1	351,789	0.3
2025	2	343,555	0.6

### 3.2 Clinical presentation

At the time of first hospital admission, the most common presenting features were progressive, symmetric proximal muscle weakness of the lower limbs (70.6%), generalized weakness (70.6%), and characteristic skin manifestations (58.8%). Heliotrope rash and Gottron’s papules were each observed in 47.1% of children with JDM.

Musculoskeletal symptoms such as myalgia or muscle tenderness were reported in 35.3% of children, while joint pain occurred in 47.1%. Functional impairment affecting daily activities was documented in 23.5% of children. Dysphagia was not observed at presentation.

Extramuscular involvement was uncommon. One child with JDM (5.9%) presented with respiratory symptoms and was subsequently diagnosed with interstitial lung disease. One child had calcinosis. Macrophage activation syndrome and mortality were not observed during the study period. Detailed clinical features are summarized in Table 2.

**Table 2:** Clinical features and selected extramuscular manifestations at initial hospital presentation among 17 children with juvenile dermatomyositis (JDM).

Clinical feature	Percentage of children with JDM
Fever	23,5% (4/17)
Weakness	70,6% (12/17)
Skin rash	58,8% (10/17)
Myalgia, tenderness	35,3% (6/17)
Joint pain	47,1% (8/17)
Functional impairment	23,5% (4/17)
Progressive, symmetric proximal muscle weakness (legs)	70,6% (12/17)
Progressive, symmetric proximal muscle weakness (arms)	41,2% (7/17)
Progressive, symmetric proximal muscle weakness (neck)	29,4% (5/17)
Dysphagia	0% (0/17)
Respiratory problems	5,9% (1/17)
Interstitial lung disease	5,9% (1/17)
Heliotrope rash (eyelids)	47,1% (8/17)
Gottron’s papules (knuckles/joints)	47,1% (8/17)
Photosensitivity	5,9% (1/17)
Calcinosis	5.9% (1/17)
Raynaud’s phenomenon	5,9% (1/17)

### 3.3 Laboratory findings

Laboratory markers, reference ranges, and proportions of abnormal results among children with JDM at hospital admission are summarized in Table 3; not all parameters were available for every child.

**Table 3:** Laboratory markers, reference ranges, and numbers of abnormal results among children with juvenile dermatomyositis (JDM) at hospital admission.

Laboratory marker	Reference range	Abnormal/total
CPK, U/L	20–250	6/17
LDH, U/L	100–225	16/17
ALP, IU/L	30–130	4/10
ALT, U/L	13–45	9/17
AST, U/L	10–50	9/17
CRP, mg/L	<10	0/17
ESR, mm/h	<2	16/17
ANA	<1:40	8/17
SAA, mg/L	3–10	4/6
RF, U/mL	<20	1/16

CPK, creatine phosphokinase; LDH, lactate dehydrogenase; ALP, alkaline phosphatase; ALT, alanine aminotransferase; AST, aspartate aminotransferase; CRP, C-reactive protein; ESR, erythrocyte sedimentation rate; ANA, antinuclear antibodies; SAA, serum amyloid A; RF, rheumatoid factor. Units are shown in the table.

Elevated serum lactate dehydrogenase (LDH) was the most frequent laboratory abnormality, observed in 16 of 17 children (94%). LDH levels were above the reference range in most children. Elevated erythrocyte sedimentation rate (ESR) was also common, detected in 16 of 17 children (94%).

Creatine phosphokinase (CPK) levels were elevated in 6 of 17 children (35%). Alanine aminotransferase (ALT) and aspartate aminotransferase (AST) elevations were observed in approximately half of the cohort. C-reactive protein (CRP) values remained within the normal range in all children at presentation (Table 3).

Antinuclear antibodies (ANA) were detected in 8 of 17 children (47.1%), with titers ranging from 1:80 to 1:640. Rheumatoid factor (RF) positivity was identified in one child. Despite variable elevations of muscle enzymes, reduced muscle strength assessed using the manual muscle testing 8 (MMT8) score was documented in the majority of children, underscoring the limited correlation between biochemical markers and clinical muscle involvement in JDM (Table 4).

**Table 4:** Individual laboratory findings at hospital admission among 17 children with juvenile dermatomyositis (JDM), presented by study participant and laboratory parameter.

Participant	CPK (U/L)	LDH (U/L)	ALP (IU/L)	ALT (U/L)	AST (U/L)	CRP (mg/L)	ESR (mm/h)	ANA	SAA (mg/L)	RF (U/mL)	HLA-B27	MMT8 (points)
P1	76	554	986	65	34.6	0.8	2	ND	3.2	4.8	ND	38
P2	43	144	87	13.2	14.2	2.6	28	1:640	–	0	ND	–
P3	3,295	788	61	70.6	162.8	0.47	24	ND	–	6.5	ND	41
P4	4,054	702	–	103.8	200.9	2.45	23	ND	N	ND	ND	68
P5	550	417	–	119.46	142.6	0.85	15	–	–	8.6	ND	–
P6	57	547	118	22.2	47.5	0.13	43	1:320	3.5	12.1	ND	70
P7	225	960	64	158.7	206.0	2.67	17	1:80	5.9	5.2	ND	80
P8	12,982	1,367	–	161.08	217.5	5.70	66	–	0.5	–	ND	55
P9	328	719	88	31.30	80.17	2.72	23	1:80	–	ND	ND	55
P10	42	451	132	9.9	40.5	0.91	25	1:320	–	6.8	ND	74
P11	208	466	118	16.5	52.3	0.64	53	–	–	5.0	ND	33
P12	160	305	–	15.3	30.6	0.14	20	–	–	9.7	ND	80
P13	102	246	–	9.9	23.8	0.4	4	1:80	–	9.9	ND	80
P14	115	387	127	13.8	50.3	0.38	43	1:160	3.5	8.8	ND	–
P15	98	353	132	30.7	36.9	0.67	33	–	35.1	8.8	ND	–
P16	4,978	942	125	159.9	205.0	0.79	10	–	–	394.9	ND	70
P17	183	441	–	31.1	43.8	0.52	19	1:80	–	–	ND	80

P1–P17, individual study participants; CPK, creatine phosphokinase; LDH, lactate dehydrogenase; ALP, alkaline phosphatase; ALT, alanine aminotransferase; AST, aspartate aminotransferase; CRP, C-reactive protein; ESR, erythrocyte sedimentation rate; ANA, antinuclear antibodies; SAA, serum amyloid A; RF, rheumatoid factor; HLA-B27, human leukocyte antigen B27; MMT8, manual muscle testing 8; ND, not detected; N, normal; –, not available. Units are shown in the table.

Specific autoantibodies associated with systemic autoimmune rheumatic diseases (SARD) and autoimmune myopathies were detected in 10 of 17 children. Five children were not tested for specific autoantibodies because these assays were unavailable during the earlier study period. In two children, all tested autoantibodies were negative.

Among the identified autoantibodies, anti–TIF1*γ* antibodies were detected in five children. Anti–MDA5 antibodies were present in one child. Anti–PM-Scl75 antibodies were detected in two children, and anti–PM-Scl100 antibodies in one child. Anti–SRP antibodies were identified in one child. Anti–Ro-52 antibodies were present in one child. Anti–NXP2 antibodies were detected in two children. Anti–Mi-2*α* antibodies were found in one child, and anti–cN-1A and anti-OJ antibodies were each detected in one child. The distribution of myositis-specific and myositis-associated autoantibodies is shown in Table 5.

**Table 5:** Distribution of myositis-specific and myositis-associated autoantibodies detected among 17 children with juvenile dermatomyositis (JDM).

Autoantibody	Number of children with JDM, *n*	Percentage, %
Anti-TIF1*γ*	5	29.4
Anti-MDA5	1	5.9
Anti-PM-Scl75	2	11.8
Anti-PM-Scl100	1	5.9
Anti-SRP	1	5.9
Anti-Ro-52	1	5.9
Anti-NXP2	2	11.8
Anti–Mi-2*α*	1	5.9
Anti-cN-1A	1	5.9
Anti-OJ	1	5.9

Anti-TIF1γ, anti–transcription intermediary factor 1-gamma; anti-MDA5, anti–melanoma differentiation-associated protein 5; anti-PM-Scl75/100, anti–polymyositis/scleroderma complex 75/100-kDa subunits; anti-SRP, anti–signal recognition particle; anti-Ro52, anti-Ro52 (TRIM21); anti-NXP2, anti–nuclear matrix protein 2; anti-Mi-2α, anti-Mi-2 alpha; anti-cN-1A, anti–cytosolic 5′-nucleotidase 1A; anti-OJ, anti–isoleucyl-tRNA synthetase antibody.

These findings demonstrate considerable heterogeneity in autoantibody profiles among children with JDM, with anti–TIF1*γ* antibodies being the most prevalent. The absence of testing in several children and negative serology in others highlights the limitations of serological diagnostics and suggests that a proportion of children may remain seronegative.

The heterogeneity of autoantibody profiles observed in our cohort is consistent with previous reports, in which no single antibody is universally present or diagnostic (5). Anti–TIF1*γ* was the most frequently detected antibody in our cohort. We also identified anti–MDA5, anti–NXP2, anti–Mi-2, and anti–SRP antibodies, which have been associated with distinct clinical phenotypes in JDM (5). In our small cohort, these associations were not always clearly evident, likely because of the limited sample size. Several children were either not tested or remained seronegative, reflecting real-world limitations in assay availability and reinforcing that autoantibody testing, while clinically useful, is not required for diagnosis.

### 3.4 Radiologic and instrumental findings

Radiologic evaluation was performed selectively based on clinical indication. When performed, magnetic resonance imaging (MRI) of proximal muscles demonstrated increased T2 signal intensity and muscle edema consistent with active inflammatory myopathy. These findings supported the clinical diagnosis when laboratory enzyme elevations were inconclusive.

Chest radiography was performed in all children with JDM before initiation of immunosuppressive therapy. One child demonstrated radiologic features consistent with interstitial lung disease and subsequently underwent computed tomography (CT), which influenced therapeutic decisions. No other significant pulmonary abnormalities were identified.

Muscle biopsy and electromyography were not routinely performed, in accordance with EULAR/ACR recommendations, because diagnosis was established based on clinical, laboratory, and imaging findings (1, 3).

### 3.5 Treatment and disease course

All children received pharmacological therapy. Initial treatment most commonly consisted of systemic glucocorticosteroids (82.4%) in combination with non-steroidal anti-inflammatory drugs (82.4%). Methotrexate was used as the primary steroid-sparing agent in 76.6% of children and represented the cornerstone of long-term immunosuppressive therapy. Four children received intravenous immunoglobulin; in one child, treatment was discontinued because of pronounced parenchymal lung complications.

Additional conventional immunosuppressive agents, including cyclosporine (17.6%), hydroxychloroquine (11.8%), mycophenolate mofetil (5.9%), and cyclophosphamide (5.9%), were introduced in cases of persistent disease activity or organ involvement.

Biologic therapy was used sparingly. One child received rituximab because of interstitial lung disease, and one received a TNF-α inhibitor following disease relapse. The latter subsequently developed intestinal vasculitis, necessitating discontinuation of biologic therapy and transition to mycophenolate mofetil. Pharmacological treatments are summarized in Table 6.

**Table 6:** Pharmacological treatments received by 17 children with juvenile dermatomyositis (JDM) during the study period.

Pharmacological therapy	Children with JDM, *n* (%)
NSAIDs	82.4% (14/17)
GCS	82.4% (14/17)
MTX	76.6% (13/17)
AZA	0% (0/17)
CsA	17.6% (3/17)
MMF	5.9% (1/17)
Rituximab	5.9% (1/17)
TNF-α inhibitors	5.9% (1/17)
JAK inhibitors	0% (0/17)
IVIG	23.5% (4/17)
HCQ	11.8% (2/17)
CYC	5.9% (1/17)

NSAIDs, non-steroidal anti-inflammatory drugs; GCS, glucocorticosteroids; MTX, methotrexate; AZA, azathioprine; CsA, cyclosporine; MMF, mycophenolate mofetil; IVIG, intravenous immunoglobulin; HCQ, hydroxychloroquine; CYC, cyclophosphamide; TNF, tumor necrosis factor; JAK, Janus kinase. Percentages are calculated from the total cohort (*n* = 17).

Remission was achieved in 70.6% of patients with within the first year using conventional therapy alone. At three years after disease onset, 23.5% of patients remained on ongoing pharmacological treatment. The most severe complication was duodenal perforation associated with intestinal vasculitis; treatment included cyclophosphamide. No fatal outcomes were recorded. The duration of therapy prior to medication discontinuation is presented in Table 7.

**Table 7:** Number of children with juvenile dermatomyositis (JDM) who discontinued each medication within specified treatment intervals.

Treatment duration	Number of children with JDM who discontinued each medication within the specified treatment interval						
	MTX	GCS	CsA	MMF	HCQ	CYC	Biologic
<1 year	3	6			1	1	2
1–2 years	3	5	1	1			
2–3 years		2					
>3 years	2	1	1				

MTX, methotrexate; GCS, glucocorticosteroids; CsA, cyclosporine; MMF, mycophenolate mofetil; HCQ, hydroxychloroquine; CYC, cyclophosphamide; Biologic, biologic therapy.

## 4 Discussion

This retrospective study provides the first comprehensive overview of JDM in children in Latvia, covering 2010–2025. The observed mean annual incidence of 0.32 per 100,000 children is consistent with rates reported in other European cohorts and registry populations, reflecting the rarity of this autoimmune myopathy (6, 7). The slight female predominance (female-to-male ratio, 1.4:1) and peak onset in early school age are also consistent with international data, although the small sample size limits direct comparison.

Seasonal variation in disease onset was notable, with the highest frequency in winter (52.9%). This observation may be compatible with previous hypotheses implicating environmental triggers or infections in JDM pathogenesis, although our study was not designed to determine causality.

The median age at diagnosis of six years is comparable to other cohorts of children with JDM (6, 7). The median duration from symptom onset to diagnosis (90 days) highlights the potential for diagnostic delay in a rare disease, despite access to a national tertiary referral center.

Clinically, children with JDM presented with proximal muscle weakness, exercise-induced fatigue, and characteristic rashes, including heliotrope rash and Gottron’s papules. These findings support the clinical utility of the 2017 EULAR/ACR classification criteria, which can be applied without mandatory muscle biopsy (1, 3). In children presenting with proximal weakness, distinguishing JDM from muscular dystrophy remains an important diagnostic consideration (8). Laboratory assessment revealed frequent elevations in LDH (94%) and ALT (53%), whereas CPK elevation was less consistent (35%). This difference between biochemical markers and functional impairment, as reflected by the MMT8 score, highlights the importance of integrating clinical and laboratory assessment when monitoring disease activity.

All children received pharmacological therapy, initially with glucocorticosteroids and/or NSAIDs. Methotrexate was the most frequently used steroid-sparing agent, and remission was achieved in 70.6% of children within the first year. Additional immunosuppressive and biologic agents, including hydroxychloroquine, cyclosporine, mycophenolate mofetil, adalimumab, rituximab, and cyclophosphamide, were used in nine children with persistent or complicated disease. One child required rituximab primarily because of lung involvement and interstitial lung disease. Another developed intestinal vasculitis while receiving adalimumab; adalimumab was discontinued and treatment was subsequently switched to cyclophosphamide. By three years after disease onset, 23.5% of children remained on pharmacological treatment. Biologic therapies have also been investigated in refractory JDM, including abatacept, supporting their role as potential options when conventional treatment is insufficient (9).

Our findings are consistent with the established approach of using glucocorticosteroids and methotrexate as the mainstay of initial treatment, with additional immunosuppressive or biologic therapy considered for persistent or complicated disease. The absence of macrophage activation syndrome (MAS) and mortality in our cohort is reassuring, although MAS has been reported in a small proportion of children with JDM (10).

In our cohort, myositis-related autoantibodies were identified in a subset of children, with anti–TIF1*γ* being the most frequently detected. The distribution of anti–MDA5, anti–NXP2, anti–Mi-2, and anti–SRP antibodies was broadly consistent with the heterogeneity described in previous studies, although phenotype–antibody associations could not be reliably assessed because of the small sample size (5). Several children were either not tested or remained seronegative, reflecting limitations in assay availability and reinforcing that autoantibody testing provides additional clinical information but is not required for diagnosis (5).

This study has several limitations. The small sample size reflects the rarity of JDM in Latvia and limits statistical power and subgroup analysis. Missing laboratory data for some markers may also have influenced interpretation of disease activity patterns. Nevertheless, the cohort represents the experience of the national tertiary pediatric referral center over 15 years and provides valuable information on JDM presentation, disease course, laboratory profiles, and treatment outcomes in a previously unreported population.

In conclusion, JDM in Latvia shows clinical, laboratory, and therapeutic patterns broadly comparable to those reported in international cohorts. Early recognition and prompt treatment with glucocorticosteroids and methotrexate were associated with remission in most children within the first year, while immunosuppressive and biologic therapies were reserved for persistent or complicated disease. LDH was frequently elevated in this cohort and may be a useful marker to consider alongside clinical assessment. Overall, long-term outcomes were favorable with contemporary management strategies.

## 5 Conclusions

This retrospective study covering 2010–2025 shows that JDM in Latvia generally presents similarly to what has been described in other cohorts of children. In our cohort, proximal muscle weakness and characteristic skin findings were the most consistent clinical features, while LDH and ESR were the most frequently elevated laboratory markers. Most children responded well to early treatment with glucocorticosteroids and methotrexate, with remission achieved in the majority within the first year and only rare need for biologic therapy. Muscle MRI was particularly helpful in selected cases, especially when laboratory findings were inconclusive. Overall outcomes were favorable, with few severe complications and no mortality. From a practical standpoint, this study provides a nationwide snapshot of JDM in Latvia and emphasizes the importance of early clinical recognition, integrated assessment beyond laboratory markers, and prompt treatment.

## Data Availability

The raw data supporting the conclusions of this article will be made available by the authors, without undue reservation.

## References

[1] Bottai M Tjärnlund A Santoni G Werth VP Pilkington C de Visser M. EULAR/ACR classification criteria for adult and juvenile idiopathic inflammatory myopathies and their major subgroups: a methodology report. RMD Open. (2017) 3(2):e000507. 10.1136/rmdopen-2017-000507 29177080 PMC5687535

[2] Chan Ng PP Syntakas AE Wedderburn LR. Juvenile dermatomyositis: new insights into pathogenesis and clinical applications. Pediatr Rheumatol. (2025) 23(1):115. 10.1186/s12969-025-01146-8 PMC12613612 41233908

[3] Lundberg IE Tjärnlund A Bottai M Werth VP Pilkington C de Visser M. 2017 European league Against Rheumatism/American College of Rheumatology classification criteria for adult and juvenile idiopathic inflammatory myopathies and their major subgroups. Ann Rheum Dis. (2017) 76(12):1955–64. 10.1136/annrheumdis-2017-211468 29079590 PMC5736307

[4] Covert LT Patel H Osman A Duncan L Dvergsten J Truskey GA. Effect of type I interferon on engineered pediatric skeletal muscle: a promising model for juvenile dermatomyositis. Rheumatology. (2024) 63(1):209–17. 10.1093/rheumatology/kead186 37094222 PMC10765138

[5] Kwiatkowska D Reich A. The significance of autoantibodies in juvenile dermatomyositis. Biomed Res Int. (2021) 2021:5513544. 10.1155/2021/5513544 34840975 PMC8626176

[6] Moegle C Lipsker D. Dermatomyosite de l’enfant. Série descriptive de 22 cas [juvenile dermatomyositis: a series of 22 cases]. Ann Dermatol Venereol. (2020) 147(8-9):494–503. 10.1016/j.annder.2020.04.016 32532518

[7] Neely J Ardalan K Huber A Kim S, Childhood Arthritis and Rheumatology Research Alliance Investigators. Baseline characteristics of children with juvenile dermatomyositis enrolled in the first year of the new childhood arthritis and rheumatology research alliance registry. Pediatr Rheumatol Online J. (2022) 20(1):50. 10.1186/s12969-022-00709-3 35854378 PMC9295519

[8] Madison JA Ferris SP Kerski M Hile G Matossian S Komisar C. Expert perspective: diagnostic approach to differentiating juvenile dermatomyositis from muscular dystrophy. Arthritis Rheumatol. (2025) 77(5):506–20. 10.1002/art.43057 39542842 PMC12039475

[9] Curiel RV Nguyen W Mamyrova G Jones D Ehrlich A Brindle KA. Improvement in disease activity in refractory juvenile dermatomyositis following Abatacept therapy. Arthritis Rheumatol. (2023) 75(7):1229–37. 10.1002/art.42450 36657109 PMC10339445

[10] Chang Y Shan X Ge Y. Macrophage activation syndrome in juvenile dermatomyositis: a case report and a comprehensive review of the literature. Pediatr Rheumatol Online J. (2023) 21(1):106. 10.1186/s12969-023-00893-w 37735702 PMC10515226

